# Spontaneous Pneumothorax Secondary to Pulmonary Paragonimiasis: A Case Report Highlighting Diagnostic Delay in Eastern Nepal

**DOI:** 10.1002/rcr2.70131

**Published:** 2025-02-26

**Authors:** Arun Kumar Mahato, Sonu Shah, Sandip Kumar Sah, K. C. Rupak, Bigyan Adhikari, Ashit Pokhrel

**Affiliations:** ^1^ Department of Pulmonary, Critical Care and Sleep Medicine Nobel Medical College Teaching Hospital Biratnagar Nepal; ^2^ Department of Internal Medicine Nobel Medical College Teaching Hospital Biratnagar Nepal; ^3^ Nobel Medical College Teaching Hospital Biratnagar Nepal

**Keywords:** freshwater snail, hypereosinophilia, *Paragonimus westermani*, praziquantel, spontaneous pneumothorax

## Abstract

Paragonimiasis, or oriental lung fluke, a zoonotic disease acquired by consuming raw or undercooked crustaceans such as crabs or snails, can mimic tuberculosis in endemic regions. There is a wide range of clinical symptoms depending on the parasite location, from non‐specific symptoms like diarrhoea, abdominal pain, rash, and fever to pleuropulmonary symptoms like cough, haemoptysis, chest pain, and dyspnoea. Pneumothorax is the rare clinical picture. We present a case of a 48‐year‐old male non‐smoker with a persistent cough for 3 months and who was treated for acute eosinophilic pneumonia initially at another centre. He then presented with symptoms of spontaneous pneumothorax at our centre and was treated with a chest tube. Revisiting the history, raw snail consumption was noted. Microscopic examination of a wet mount bronchoalveolar lavage (BAL) confirmed the diagnosis of *Paragonimus westermani*, and praziquantel was started; he improved significantly within 48 h.

## Graphical abstract

Here we present a 48‐year‐old farmer diagnosed with pleuropulmonary paragonimiasis complicated by spontaneous pneumothorax. This case highlights the diagnostic dilemmas posed and stresses the inclusion of parasitic diseases in the differential diagnosis, especially in resource‐poor settings and tuberculosis‐endemic countries, thus avoiding anti‐tubercular treatment for non‐tubercular conditions.

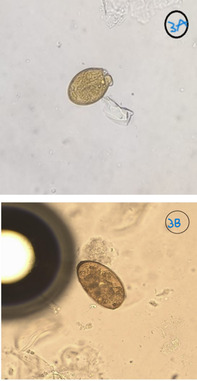

## 1 Introduction

Pulmonary paragonimiasis, or oriental lung fluke, is caused by consuming food contaminated with the species *Paragonimus westermani*. It is endemic to Southeast Asia, including Nepal. Infection occurs through consuming uncooked or undercooked, marinated, pickled freshwater crustaceans, such as crabs or snails, harbouring infective metacercaria larvae (infective stage). Following ingestion, metacercaria larvae penetrate the intestinal wall, migrate through the diaphragm into pleural spaces, and eventually reach the lung parenchyma, where they mature into adult flukes. This then results in a chronic inflammatory response, leading to cyst formation around paired adults [1]. The rupture of these cysts into the pleural cavity can produce spontaneous pneumothorax, while eggs that are expelled via cyst—bronchial fistulas either get coughed up in sputum or pass in the faeces.

Clinically, patients with paragonimiasis commonly present with pleuropulmonary and extrapulmonary symptoms; the most frequent ones include cough, haemoptysis, dyspnoea and chest pain. This often‐mimics pulmonary tuberculosis (PTB) resulting in misdiagnosis [1]. Due to the parasitic nature of the infection, systemic symptoms like fatigue and fever, coupled with eosinophilia, are common. In endemic areas, one must consider the possibility of a parasitic infection in a patient showing atypical respiratory symptoms. The disease is more common where tuberculosis is highly prevalent [2].

Here we present a 48‐year‐old farmer diagnosed with pleuropulmonary paragonimiasis (PP) complicated by spontaneous pneumothorax. This case highlights the diagnostic dilemmas posed and stresses the inclusion of parasitic diseases in the differential diagnosis, especially in resource‐poor settings and tuberculosis‐endemic countries, thus avoiding anti‐tubercular treatment for non‐tubercular conditions [3].

## 2 Case Report

We report a case of a non‐alcoholic, non‐smoker 48‐year‐old farmer without any significant past medical history, who presented to Nobel Medical College and Teaching Hospital Outpatient Department (OPD) with the chief complaint of a cough for 3 months. The cough was initially dry before becoming blood mixed. He also reported chest pain, evening rise of fever, chills and abrupt dyspnoea on exertion.

**FIGURE 1:**
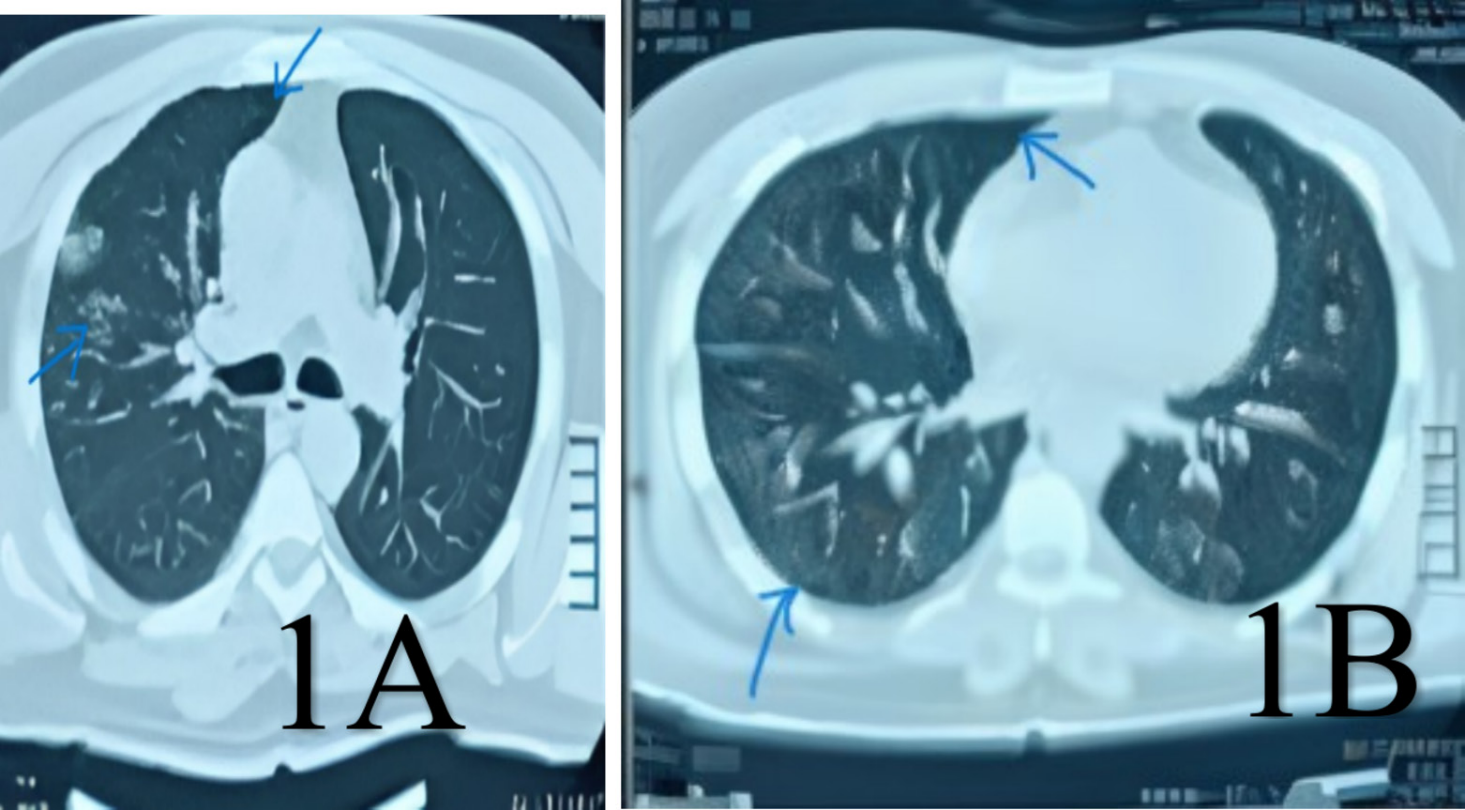
(A) and (B): CT scan of Chest showing ground glass opacity in the basal segment of the lower lobe of the left lung likely infective pathology with few fibrotic strands and a thin strip of loculated pneumothorax—3 mm along the apical region of the upper lobe of the left lung.

Upon review of his medical history, we found that he had recently been to a tertiary care centre with a similar illness, where baseline investigations revealed an absolute eosinophil count (AEC) to be markedly elevated to 2480/cu.mm (31%: normal range is < 7%), peripheral blood smear showing normal morphology of RBCs with eosinophilia, and USG abdomen and pelvis was done showing mild splenomegaly −14.4 cm in size with Grade II fatty liver. Additionally, a chest CT scan revealed a few small thick‐walled cystic bronchiectatic changes in the upper and lower lobes of the right lung and the lower lobe of the left lung, subtle peri‐cavitary consolidation, and ground‐glass opacities and a few fibrotic threads in the basal section of the left lung's lower lobe (Figure 1A,B). After these findings, parasitic‐induced eosinophilic pneumonia (Loeffler syndrome), drug or toxin induced eosinophilic pneumonia, tropical pulmonary eosinophilia, allergic granulomatosis, asthma/allergy, fungal (esp. *Aspergillus*, *Pneumocystis*), and tuberculosis were considered as the differentials of hypereosinophilia with pulmonary infiltrates. A provisional diagnosis of acute eosinophilic pneumonia (AEP) was made, and the patient was prescribed antihistaminic, low‐dose steroids, antibiotics, rota halers, and proton pump inhibitors for 2 weeks. Despite treatment, he could not get full relief from cough, dyspnoea and chest pain. He visited that centre again after a few days. His blood investigation showed raised erythrocyte sedimentation rate (ESR), normal white blood cell (WBC), and normal eosinophil count; then his sputum sample was sent for acid‐fast bacilli (AFB) and Gene Xpert due to the resemblance of his symptoms to PTB. However, the reports of AFB and Gene Xpert were negative.

**FIGURE 2:**
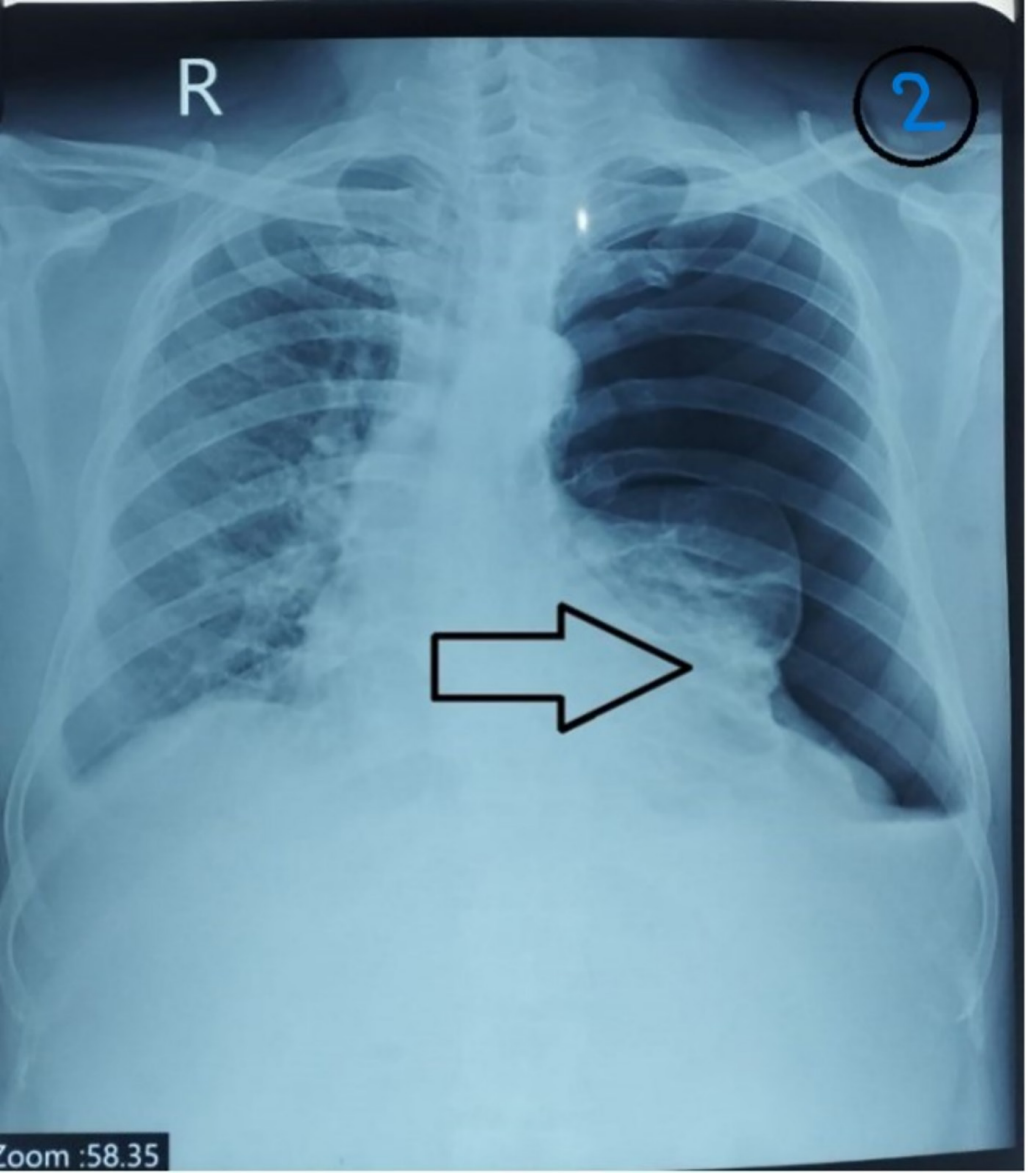
Chest X‐ray showing left‐sided pneumothorax shown by arrow.

He then visited our centre after 3 months of his illness in the pulmonology outpatient department (OPD) with symptoms of dyspnoea on exertion and chronic dry cough. Furthermore, clinical examination revealed hyper resonance on percussion and diminished breath sounds on the left side of the chest, which was supported by a chest X‐ray that showed a left‐sided pneumothorax of massive size (Figure 2). The patient was therefore directly admitted to the pulmonary ward for inserting a chest tube and kept on supplemental oxygen, after which dyspnoea showed significant improvement. A CT scan of the chest confirmed left‐sided pneumothorax, excluding other diagnoses.

**FIGURE 3:**
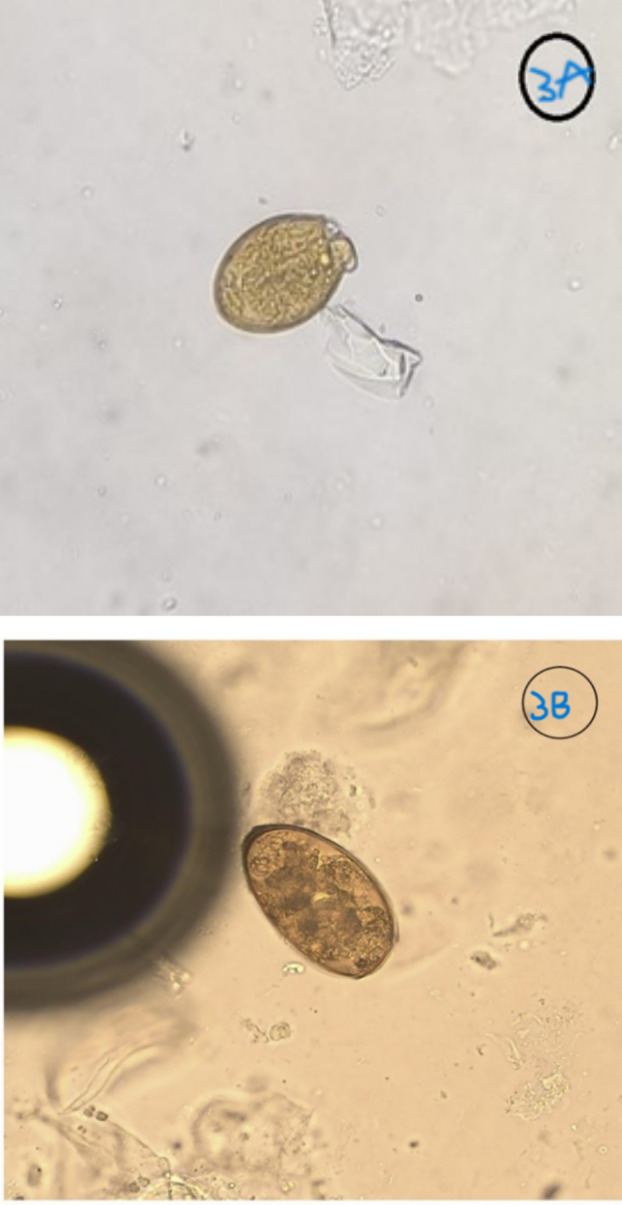
(A) BAL wet mount showing eggs of *Paragonimus westermani*. (B) Sputum Smear showing the same egg as *Paragonimus westermani*.

On detailed interrogation about his dietary habits, he reported consuming raw freshwater snails to treat joint pain, a practice common in his community for managing joint pain. Then, the provisional diagnosis of PP was revised after excluding tuberculosis, granulomatous polyangiitis, drug or toxin‐induced eosinophilic pneumonia and aspergillosis. Following stabilisation, a bronchoscopy was planned, which showed blood clots in the upper lobe bronchus. On repeat, microscopic examination of the wet mount of bronchoalveolar lavage (BAL) revealed a rare finding of an egg of *Paragonimus* species (Figure 3A) along with Gram‐negative bacilli and Gram‐positive cocci. Sputum examination also showed a yellow‐brown ovoid structure with an operculum resembling the *Paragonimus* egg (Figure 3B). The patient was diagnosed with PP and treated with praziquantel (25 mg/kg three times daily) for 3 days. The clinical symptoms subsided gradually and he was discharged after 5 days with dietary counselling and follow‐up instructions after 1 month. During the last follow‐up, there was no recurrence of pneumothorax or pulmonary abnormalities on imaging. He was symptom‐free.

## 3 Discussion

Paragonimiasis is a food‐borne zoonotic infection caused by trematodes of the genus *Paragonimus*. With an estimated global burden of 22.8 million, this condition predominantly affects Southeast Asia, including Nepal. China has the most case reports, reporting about 425 cases; Nepal reported 6 cases prior, India reported 4 cases, while the western part of the world, like the United States, had reported 12 cases [1, 3, 4]. *Paragonimus westermani* is the most common type prevalent in this region, including Nepal. *P. heterotremus* has also been detected recently [4]. The major aspect of the case is that pneumothorax is an uncommon but serious complication of PP, a condition often mistaken for tuberculosis.

**FIGURE 4:**
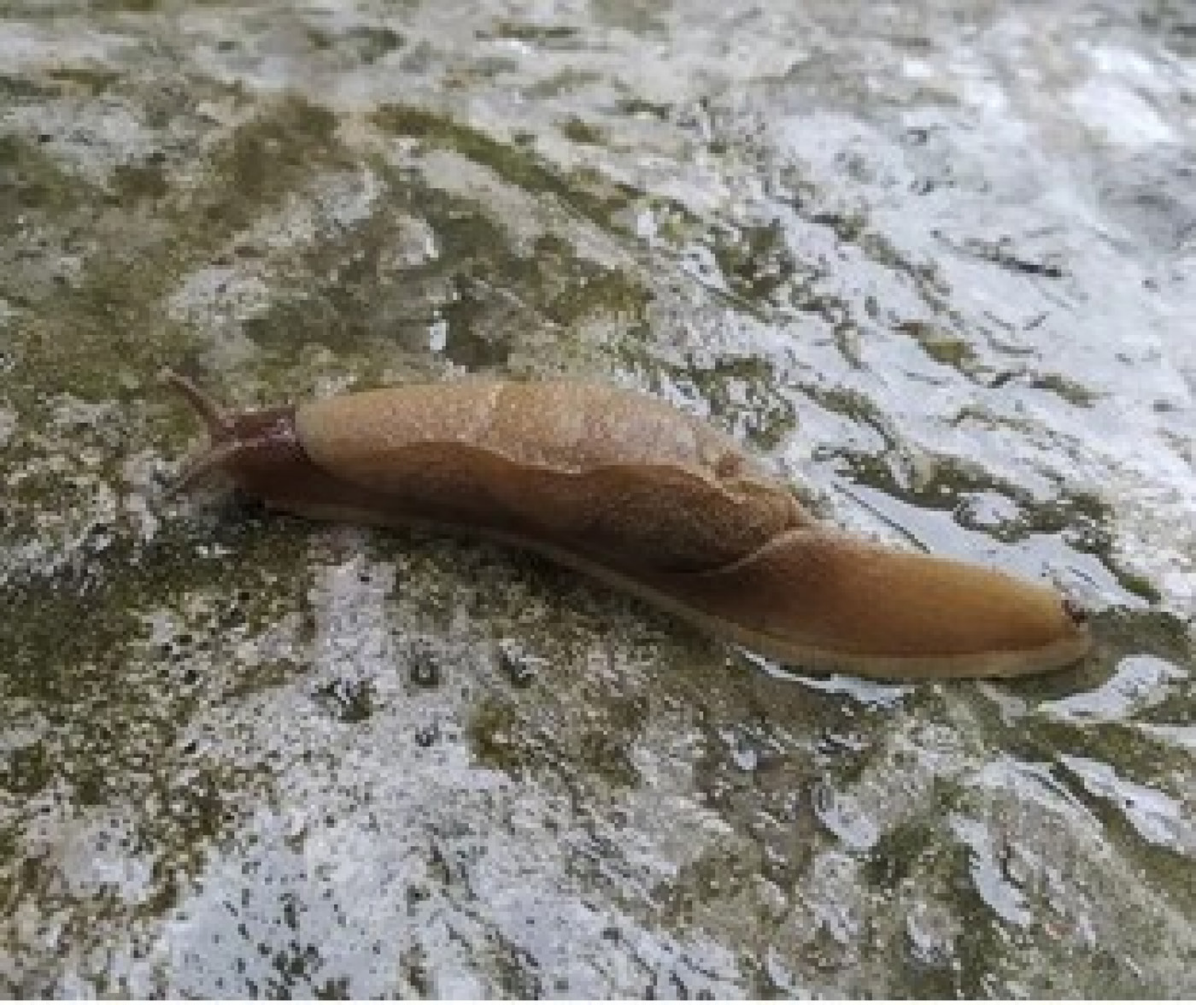
Consumption of Snail (Chiple Kira), also known as Leopard slug is a very common traditional medicine in the eastern part of Nepal.

The life cycle of Paragonimus is completed in the three hosts: snail, crustacean and hhuman. Human beings are the tertiary, definitive hosts who get infected by eating uncooked, undercooked, pickled or marinated snails or crustaceans like crabs (Figure 4) [1]. Once ingested, larvae penetrate the intestinal wall, enter through the diaphragm into pleural spaces, and eventually migrate to the lung parenchyma, where they mature into adult flukes. Cyst formation around paired adults occurs due to the chronic inflammatory response [1, 4]. The rupture of these cysts into the pleural cavity can produce spontaneous pneumothorax [5]. There is no human‐to‐human transmission.

Symptoms of paragonimiasis depend on the worm's location. Common manifestations include productive cough with bloody sputum (77.9%), nonproductive cough (1.5%), and chest pain (67.6%) [6]. However, these symptoms often mimic those of PTB, leading to diagnostic delays. For instance, haemoptysis, a hallmark of paragonimiasis, can be mistaken for smear‐negative PTB, as reported in many studies. The clinical parallels between paragonimiasis and tuberculosis frequently delay diagnosis, especially in TB‐endemic regions like Nepal.

Our patient was initially misdiagnosed with hypereosinophilic syndrome and treated with corticosteroids, reflecting the diagnostic pitfalls of this condition. Similarly, other studies have documented that PP may be misdiagnosed as unresolving pneumonia or lung cancer [4, 7].

The complete blood counts in Paragonimiasis typically show leukocytosis and eosinophilia (75.5%) [4, 6] however, in our case, the normal eosinophil count was an odd finding. This might have been possible due to the use of steroids before for eosinophilic lung disease—AEP in our case. Radiologically, pleural effusion (47%) is the most common manifestation of paragonimiasis, followed by pneumothorax (16.9%), nodular opacities (11.5%), and airspace consolidations (6.5%) [4, 6]. Pneumothorax is observed as an early radiologic finding in 16.9% of cases [6]. The presence of pleural manifestations highlights the need for early suspicion of paragonimiasis in endemic areas [6]. In our patient, subtle clinical signs including hyper‐resonance and diminished breath sounds, along with imaging studies, revealed pneumothorax without effusion. The treatment was managed according to guidelines for secondary spontaneous pneumothorax, including chest tube drainage.

Definitive diagnosis of paragonimiasis relies on identifying Paragonimus eggs in sputum, faeces, BAL fluid or pleural fluid. However, egg detection rates are low, ranging from 28% to 38%, necessitating repeated testing [5]. Serological tests offer high sensitivity and specificity [5], and if available, multiple dot enzyme‐linked immunosorbent assay (ELISA) should always be considered for patients with eosinophilia and pulmonary findings like nodularity, opacities and effusion. In resource‐limited countries like Nepal, where sophisticated tests are not readily available, a high index of suspicion for paragonimiasis is crucial, particularly in patients with a history of snail intake. In our case, Paragonimus ova were identified in BAL and sputum after several tests, confirming the diagnosis. Failure to obtain a positive sputum sample delayed diagnosis in our case.

As parasitic infections are often not considered until there is a confirmed history of exposure, a thorough dietary and travel history is critical to raise suspicion towards paragonimiasis. Correlating clinical history—including dietary habits and travel—with imaging and laboratory findings is critical in establishing the diagnosis.

The WHO recommends praziquantel (25 mg/kg three times daily for 3 days) or triclabendazole as the standard treatment for paragonimiasis. Praziquantel has an 80%–90% cure rate and a low recurrence rate. In our case, prompt praziquantel administration led to rapid recovery.

Paragonimiasis is believed to be fairly common in Nepal, with certain communities having the traditional practice of eating snails, crabs and crayfish for medicinal or social reasons. These are also being explored as cultural food in present days and thus some restaurants are providing them to tourists visiting the endemic terai region of Nepal. The problem of Paragonimiasis is thus likely to rise and continue. However, only a handful of cases (one case series and two case reports) have been reported mainly in the western and central parts of Nepal where the consumption of crabs has been reported, whereas we report the case with consumption of snails for traditional treatment of chest pain [2, 3]. As per the global TB report 2023, Nepal accounts for an estimated 70,000 TB cases. Since paragonimiasis and TB have clinical overlaps, many cases of paragonimiasis are misdiagnosed and therefore mistreated as tuberculosis [7, 8]. This leads to extended morbidity and loss of time and money in the treatment of these patients in addition to the side effects of antitubercular drugs.

This case report underlines the necessity of awareness of dietary risks associated with raw crustacean consumption among the public and consideration of parasitic infections in differential diagnoses for chronic respiratory symptoms in co‐endemic regions. Early recognition and accurate diagnosis can prevent complications such as pneumothorax.

In conclusion, paragonimiasis often mimics unresolving pneumonia, TB, aspergillosis, or other conditions, emphasising the importance of considering parasitic infections in differential diagnoses. Knowledge of local customs and dietary habits can provide crucial diagnostic insights, as was evident in our case. Early recognition and accurate diagnosis can prevent complications such as pneumothorax and reduce patients' morbidity. Awareness of dietary practices, particularly the consumption of raw or undercooked snails, is crucial. This instance emphasises the importance of raising awareness and developing diagnostic tools for parasite illnesses in endemic locations.

The patient was relieved by the resolution of symptoms after the correct diagnosis and treatment. He acknowledged the risks associated with uncooked or undercooked snail consumption.

## Author Contributions

**Arun Kumar Mahato:** conceptualisation, project administration, supervision, writing – review and editing. **Sonu Shah:** conceptualisation, project administration, supervision, writing – original draft, writing – review and editing. **Sandip Kumar Sah:** formal analysis, writing – original draft, writing – review and editing. **K. C. Rupak:** supervision, writing – original draft, writing – review and editing. **Bigyan Adhikari:** writing – original draft, writing – review and editing. **Ashit Pokhrel:** writing – review and editing.

## Ethics Statement

Written informed consent was obtained from the patient for their anonymised information to be published in this article.

## Conflicts of Interest

The authors declare no conflicts of interest.

## Data Availability

The data that support the findings of this study are available from the corresponding author upon reasonable request.
